# Commentary: Combating Sale of Counterfeit and Falsified Medicines Online: A Losing Battle

**DOI:** 10.3389/fphar.2017.00909

**Published:** 2017-12-11

**Authors:** Baobin Huang, Mingzhe Xu

**Affiliations:** Department of General Affairs, National Institutes for Food and Drug Control, Beijing, China

**Keywords:** fake medicines, falsified medicines, counterfeit medicines, supplementary testing methods, drug legislations

## Introduction

An article entitled “Combating Sale of Counterfeit and Falsified Medicines Online: A Losing Battle”, authored by Kah Seng Lee and colleagues, presented the cases of some countries, including China's fight against counterfeit and falsified medicines (CFMs). Regarding to the formulation and enforcement of legislations in this aspect in China, we comment on the article by providing further information as follows.

## China has systematic legislations in place to fight CFMs

**Figure 1:**
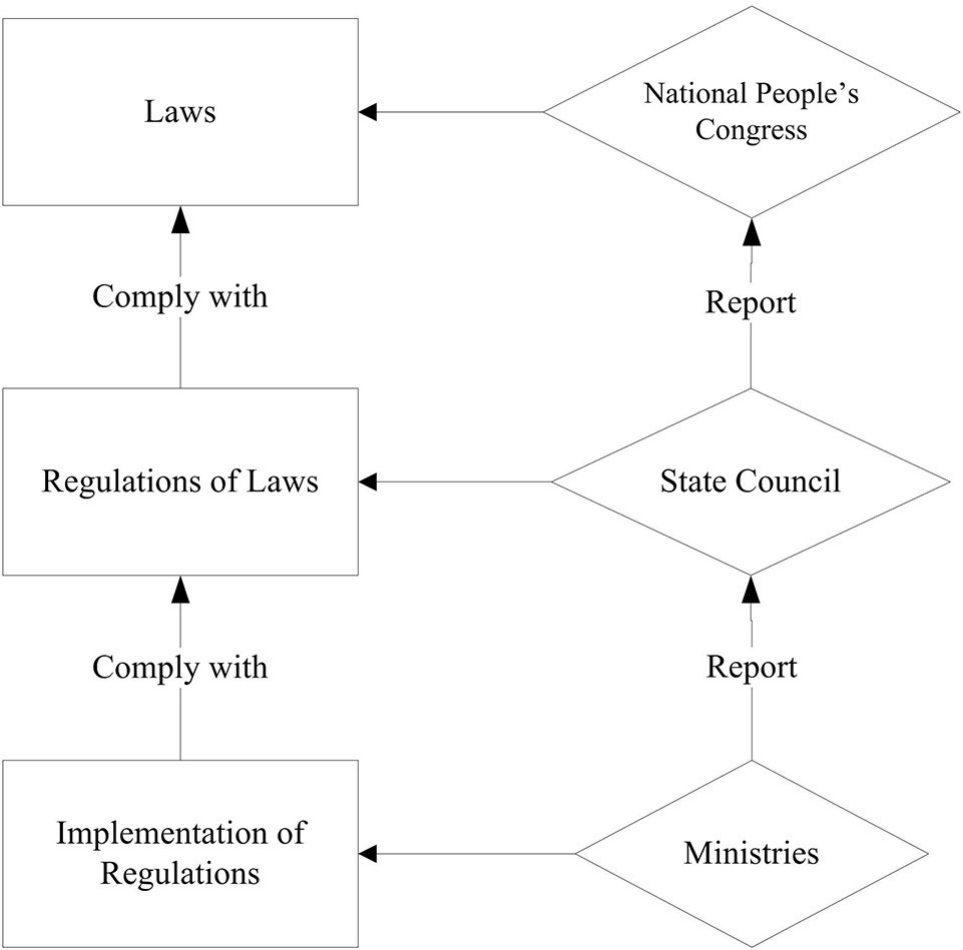
Legislations structure in China.

The authors (page 2) argue that China had previously “either no specific or weak laws” to regulate CFMs. Perhaps, they overlooked the holistic legislation system that has been in place for more than a decade. China has a three-level legislation system consisting of law, regulations on the enforcement of the law, and implementation of regulations (refer to Figure 1). In addition, judicial interpretations are issued as needed to guide the enforcement of legislations.

The Drug Administration Law, which came into effect on December 1, 2001, defines the scope of CFMs (Article 48), and states the penalties to both entities (Article 74) and responsible persons (Article 76) who produce and distribute CFMs, and to those persons or entities who provide logistics services (Article 77) (China Medicine Science Technology Press, 2008).

The Regulations on the implementation of Drug Administration Law, which took effect on September 15, 2002, recommends the application of Supplementary Testing Methods (STMs) (Article 58) (China Medicine Science Technology Press, 2008). The STMs expands upon the capability of routine testing standards to detect adulterated medicines. To implement the specific article, China's drug regulatory authority—China Food and Drug Administration (CFDA)—released notices to standardize the development and approval of STMs in 2003, and apply STMs in post-marketing surveillance in 2006 (China Food Drug Administration, 2006; Huang et al., 2014). Over the past decade, more than 220 STMs, developed by approximately 400 official medicine quality control laboratories nationwide, have been approved. These STMs have played a critical role in detecting CFMs categorized as “adulteration with chemical substances or industrial dyes,” and “use of unapproved or changed excipients without notifying regulatory body in advance.” As an effective post-market surveillance tool, the CFDA has been implementing the national medicines sampling and testing program since 1990. Over the past 3 years, 54,619 samples of different batches of medicines were tested nationwide, and the average rate of compliance with official quality standards reached to 96.0%. It demonstrates the relatively stringent quality assurance system for medicines (China Pharmaceutical News, 2017). To ensure the availability of quality-assured medicines in hospitals, China establishes essential medicines system and adopts quality-oriented procurement mechanisms in hospitals. In 2016, 683 batches of medicines sampled from different hospitals were tested, with the rate of compliance with official quality standards reaching to 96.6% (National Institutes for Food Drug Control, 2017).

A judicial interpretation on handling criminal cases concerning CFMs was issued in 2009 and updated in 2014 for effective enforcement of the law and regulations (Supreme People's Court and Supreme People's Procuratorate of P.R.C., 2009, 2014). The interpretation provides a list of conditions that deserve stringent punishment, including production and distribution of CFMs. Its focus was medicines for maternity patients, newborn babies, children, and patients with severe conditions, and it encompassed other medicines used during emergency conditions such as natural disasters and public health events.

High price is one of the factors facilitating the occurrence of CFMs (World Health Organization, 1999). To address this problem, China is promoting the application of generic medicines. To boost the confidence of healthcare professionals in locally manufactured generics, China has issued new policy to re-evaluate the quality and efficacy of essential medicines stipulating that all the listed 289 should be completed by the end of 2018 (Huang et al., 2017).

## China has adopted a multi-pronged strategy to combat illegal online drug sales

We agree with the authors (page 2) that new legislations are required to address the problem of CFMs—in particular, online sales. However, the authors failed to provide adequate information about the situation in China. In fact, China has taken a multi-pronged approach to regulate the online distribution of medicines.

First, the CFDA has set up a gate-keeping mechanism to review the qualifications of drug-related information providers (websites) and online traders of medicines (China Food Drug Administration, 2004, 2005). Second, besides the crosscutting campaign “two strikes, two setups” mentioned by the authors, an inter-agency cooperation and coordination mechanism was set up in 2015 to intensify communications between administrative law enforcement and criminal justice with regard to clue notifications, cases transfers, and information sharing (China Food and Drug Administration, 2015a). Furthermore, in January 2017, the General Office of Chinese Cabinet issued a new policy to further improve the whole chain of regulation concerning the production, distribution and consumption of CFMs, and strictly combat criminal behaviors in producing and selling CFMs (General Office of State Council, 2017). Third, to raise public awareness on the risks of purchasing medicines online, the CFDA sets up a section on its portal to release “safety alerts” and expose illegal websites periodically (China Food Drug Administration, 2013).

With systematic legislations and concerted efforts, China has made considerable progress in combating CFMs. Between 2010 and 2015, public security departments nationwide cracked down on 46,000 cases threatening to endanger drug safety. In 2014, totally 7,520 CFMs cases, valued nearly 170M RMB, were investigated and prosecuted nationwide (China Food Drug Administration, 2014). In 2015, the CFDA launched a campaign to crack down on the illegal practices in the production and distribution of Ginkgo biloba extract and its preparations. As a result, 47 firms were penalized and fined nearly103M RMB, 7 responsible persons were forbidden from engaging in work related to the production and distribution of medicines (China Food and Drug Administration, 2015b). In its blueprint of medicine regulations from 2016 to 2020, China has demonstrated its determination to severely punish illegal behaviors such as the use of inferior materials, adulteration, and changes to manufacturing practices without prior approval (State Council of P.R.C., 2017).

## Author contributions

Initiated and Designed the manuscript: BH. Wrote the manuscript: BH, MX. Both authors read and approved the final version.

### Conflict of interest statement

The authors declare that the research was conducted in the absence of any commercial or financial relationships that could be construed as a potential conflict of interest.

## References

[B1] China Food Drug Administration (2004). Measures for Regulating the Provision of Drug Related Information Service Over the Internet. http://www.sda.gov.cn/WS01/CL0053/24486.html

[B2] China Food Drug Administration (2005). Provisions on Review and Approval of Internet Drug Trading Services. Available online at: http://www.sda.gov.cn/WS01/CL0060/9432.html

[B3] China Food Drug Administration (2006). Guidelines for Drug Sampling and Testing. Available online at: http://www.sfda.gov.cn/WS01/CL0844/10583.html

[B4] China Food Drug Administration (2013). Column of “Safety Alert for Purchasing Drugs Online” on the Portal. Available online at: http://www.sda.gov.cn/WS01/CL0441/

[B5] China Food and Drug Administration (2014). Yearbook of China Food and Drug Administration in 2014. Beijing: China Food and Drug Administration.

[B6] China Food Drug Administration (2015a). Notice Issued by CFDA on Penalizing the Illegal Behaviors in Producing and Distributing Ginkgo Biloba Extract and Its Preparations. Available online at: http://www.sfda.gov.cn/WS01/CL0087/134040.html.

[B7] China Food Drug Administration (2015b). Notice to Strengthen the Connection between Administrative Law Enforcement and Criminal Justice in the Area of Food and Drug Among Four Governmental Departments. Available online at: http://www.sda.gov.cn/WS01/CL0053/139961.html

[B8] China Medicine Science and Technology Press (2008). Compilation of Food and Drug Related Laws and Regulations, 2nd Edn. Beijing: China Medicine Science and Technology Press.

[B9] China Pharmaceutical News (2017). Report on the 9th Sino-US Pharmacopeia Forum.

[B10] General Office of State Council (2017). Opinions on Further Reforming and Improving the Policies on Drug Production, Distribution and Consumption. Available online at: http://www.gov.cn/zhengce/content/2017-02/09/content_5166743.htm

[B11] Huang B. Barber S. L. Xu M. Cheng S. (2017). Make up a missed lesson-New policy to ensure the interchangeability of generic drugs in China. Pharma Res Per. 5:e00318. 10.1002/prp2.318 28603636 PMC5464346

[B12] Huang B. B. Xu M. Z. Yang Q. Y. Tian X. B. Yang Y. L. Bai D. T. (2014). Review of the approved supplementary testing methods and items on identifying adulterated chemical substances in traditional Chinese medicine preparations and herbal medicines. Chin. J. Pharm. Anal. 34, 1701–1708. 10.16155/j.0254-1793.2014.09.068

[B13] National Institutes for Food Drug Control (2017). Report on National Medicines Sampling and Testing in 2016. Available online at: http://www.nifdc.org.cn/CL0882/9872.html

[B14] State Council of P.R.C. (2017). China National Drug Safety Plan in between 2016 and 2020. Available online at: http://www.sfda.gov.cn/WS01/CL0852/169745.html

[B15] Supreme People's Court Supreme People's Procuratorate of P.R.C. (2009). Judicial Interpretations Jointly Issued by the Supreme People's Court and Supreme People's Procuratorate on Criminal Cases Concerning Substandard Drug and Counterfeits. Available online at: http://www.court.gov.cn/fabu-xiangqing-68.html

[B16] Supreme People's Court Supreme People's Procuratorate of P.R.C. (2014). Judicial Interpretations on Criminal Cases Threatening Drug Safety. Available online at: http://www.chinacourt.org/article/detail/2015/01/id/1530022.shtml

[B17] World Health Organization (1999). Guidelines for the Development of Measures to Combat Counterfeit Drugs. Geneva: World Health Organization.

